# A Systems Biology Approach to Transcription Factor Binding Site Prediction

**DOI:** 10.1371/journal.pone.0009878

**Published:** 2010-03-26

**Authors:** Xiang Zhou, Pavel Sumazin, Presha Rajbhandari, Andrea Califano

**Affiliations:** 1 Department of Biomedical Informatics (DBMI), Columbia University, New York, New York, United States of America; 2 Center for Computational Biology and Bioinformatics (C2B2), Columbia University, New York, New York, United States of America; 3 Herbert Irving Comprehensive Cancer Center, Columbia University, New York, New York, United States of America; Fondazione Telethon, Italy

## Abstract

**Background:**

The elucidation of mammalian transcriptional regulatory networks holds great promise for both basic and translational research and remains one the greatest challenges to systems biology. Recent reverse engineering methods deduce regulatory interactions from large-scale mRNA expression profiles and cross-species conserved regulatory regions in DNA. Technical challenges faced by these methods include distinguishing between direct and indirect interactions, associating transcription regulators with predicted transcription factor binding sites (TFBSs), identifying non-linearly conserved binding sites across species, and providing realistic accuracy estimates.

**Methodology/Principal Findings:**

We address these challenges by closely integrating proven methods for regulatory network reverse engineering from mRNA expression data, linearly and non-linearly conserved regulatory region discovery, and TFBS evaluation and discovery. Using an extensive test set of high-likelihood interactions, which we collected in order to provide realistic prediction-accuracy estimates, we show that a careful integration of these methods leads to significant improvements in prediction accuracy. To verify our methods, we biochemically validated TFBS predictions made for both transcription factors (TFs) and co-factors; we validated binding site predictions made using a known E2F1 DNA-binding motif on E2F1 predicted promoter targets, known E2F1 and JUND motifs on JUND predicted promoter targets, and a *de novo* discovered motif for BCL6 on BCL6 predicted promoter targets. Finally, to demonstrate accuracy of prediction using an external dataset, we showed that sites matching predicted motifs for ZNF263 are significantly enriched in recent ZNF263 ChIP-seq data.

**Conclusions/Significance:**

Using an integrative framework, we were able to address technical challenges faced by state of the art network reverse engineering methods, leading to significant improvement in direct-interaction detection and TFBS-discovery accuracy. We estimated the accuracy of our framework on a human B-cell specific test set, which may help guide future methodological development.

## Introduction

Protein-DNA binding affinity is often characterized using patterns in DNA (motifs), a key step toward TFBS discovery. Computational methods [1], [2] are essential components of any motif discovery approach, but the general computational motif discovery problem remains unsolved. Motifs are currently available for less than fifteen percent of known human TFs [3], [4], and computational motif-discovery success rates are poor, with recorded sensitivity rates below 20% in general, and considerably lower than 20% for human TFs [5]. Here, we use position-weight matrix motifs (PWMs) to model TFBSs [2], [6], but motifs may take a variety of forms including words [7], [8] and regular expressions [9], [10]. We chose PWMs to summarize TFBSs because validated PWMs are available from several sources [3], [4], and because PWMs are suitable for *de novo* discovery as they provide a good tradeoff between binding site prediction accuracy and the required volume of training data needed [11]. We study a variation on the original formulation of the motif discovery problem, which was introduced by Yoseph et al. [12]. They discovered motifs that are enriched in a foreground sequence set against a control set, and the advantage of their approach was demonstrated using both regular-expression motifs and PWMs [13], [14].

Expression, binding, and cross-species conservation data have all been used to guide motif discovery methods. Co-expression with TFs was used to identify putative promoters that may contain binding sites for TFs and could then be analyzed for TFBS enrichment [15], [16], [17]. Cross-species conservation was used to identify genomic regions that are more likely to be functionally important and thus enriched with TFBSs and other regulatory elements [18], [19]. Finally, some of the most successful motif and TFBS discovery approaches use binding data and especially high-throughput chromatin immunoprecipitation (ChIP-chip and ChIP-seq) data to identify relatively short target DNA regions with high likelihood for binding-site presence [20], [21], [22]. However, due to limited antibody availability, cell-context specificity of transcriptional interaction patterns, and the associated cost, the assembly of complete binding site repertoires for the majority of TFs is not a viable option.

Here, we show that a significant improvement in TFBS discovery can be achieved by using an integrative work-flow approach we call OmniMiner. First, we use ARACNe, a proven reverse-engineering algorithm [23], [24], [25], [26], to identify higher likelihood transcriptional targets, and we demonstrate that the inferred targets are more reliable than those predicted by co-expression. Our results suggest that by using ARACNe-predicted targets we significantly improve accuracy when compared to the co-expression approach by removing false positives among high-confidence and especially among low-confidence co-expressed targets. Then, we identify cross-species conserved regions by combining linear-alignment and pattern-discovery (*alignment-free*) based approaches. Genome-alignment-based conservation [27] can guide motif discovery [28] and help identify motifs and sites for some regulators, but it may also obscure sites that are not conserved linearly as is the case with binding-site turnover. We correct for this and show that combining the two approaches leads to significant prediction improvements. Finally, we use DME, a proven deterministic motif discovery algorithm [11], [14], [20], to discover *de novo* TFBS motifs for specific TFs and their co-factors. In our experiments, the top OmniMiner *de novo* discovered motif matched a known motif for more than 15% of the TFs in our human B cell test set. OmniMiner's recall was over 30% when the criteria was expanded to include predictions where at least one of the top five motifs matched a known motif for the TF; we note that other top 5 significant motifs may describe the binding of a co-factor. In total, our results suggest that OmniMiner's performance on unaltered human promoters is better than the performance of methods described by Tompa et al. [5] on impregnated human promoters despite the fact that motif discovery in the former is widely considered to be more challenging.

To evaluate the performance improvement associated with better target selection and cross-species conservation, we assembled human promoter sets for genes predicted to be either co-expressed with or direct transcriptional targets of a representative collection of TFs. To evaluate binding site enrichment, we measured the classification accuracy of verified TRANSFAC binding motifs associated with the TF [3]. We used binding site enrichment to compare recall rates across methods and to estimate the accuracy of *de novo* discovery methods. Then, we showed that while both our target-selection and cross-species-conservation methods improve our ability to discover bona-fide TFBSs for specific TFs, the greatest improvement arises from the integration of both methods. We compared our *de novo* motif discovery approach with GibbsModule [29], a method that was recently proposed as the state-of-the-art in integration of co-expression and cross-species conservation. While OmniMiner proceeds greedily, by identifying cross-species conserved regions in each promoter and patterns common to these conserved regions across promoters of inferred targets of a given TF, GibbsModule simultaneously identifies patterns conserved across species and across promoters of inferred targets. The simultaneous approach has the potential to maximize accuracy, but we show that OmniMiner's greedy approach produces significantly better results.

To support our estimate for prediction accuracy, we biochemically validated predictions for three TFs. Sites matching a known E2F1 motif were identified as the most enriched in predicted E2F1 targets and the second most enriched in JUND targets. Our validation confirms the presence of predicted E2F1 sites in promoters of predicted E2F1 targets, and it suggests that the majority of JUND targets are occupied by both TFs, which is consistent with the predicted co-factor role for E2F1. To demonstrate the accuracy of OmniMiner's *de novo* discovery, we validated predicted BCL6 binding sites in conserved regions of promoters of BCL6-predicted targets. Finally, to demonstrate prediction accuracy using an external dataset, we tested *de novo* discovered motifs in promoters of predicted ZNF263 targets for enrichment in ZNF263-bound regions according to ChIP-seq [30]. Our analysis showed that the three best *de novo* motifs are significantly enriched in ZNF263-bound regions.

## Results

### Use of reverse-engineering methods to identify TF targets

Co-expression has been widely used to infer regulatory interactions between TFs and their targets [16], [31], [32], [33], but co-expression alone is not sufficient for determining direct interactions. Gene sets that are co-expressed with a TF are generally enriched in its targets but also contain a large proportion of non-target and indirect targets, which substantially dilute enrichment. Regulatory networks reverse engineering algorithms like ARACNe, on the other hand, attempt to use additional properties of the data to identify genes that are more likely to be direct transcriptional targets of the TFs. Specifically, ARACNe uses the Data Processing Inequality theorem of mutual information, as well as direct knowledge of TF identity, to remove candidate regulatory interactions that are likely to be of an indirect nature [24], [25]. We used ARACNe with 100 rounds of bootstrapping to construct a regulatory network from 254 human B-cell gene-expression profiles (see ARACNe Network Inference in **Materials and methods**). Since activation and repression can be mediated by distinct co-factors and binding sites [34], we concentrated strictly on targets predicted to be activated by the TF; these constitute the majority of the interactions in the reverse-engineered regulatory network and extension to repressed subsets is straightforward. As a representative TF set for performance analysis, we selected the 70 TFs with known DNA binding motifs in TRANSFAC [3] that were predicted by ARACNe to be positive regulators of at least thirty targets, thus allowing appropriate statistical power for enrichment analysis. Thirty targets is also the suggested minimum for motif discovery using DME [11]. We assembled promoter sets for each of the 70 TFs using targets predicted by ARACNe, co-expression, and co-expression*, and identified enriched TRANSFAC motifs in each of the (70×3) sets. We refer to the ARACNe-inferred promoter set as the *conservation-free set* because it is assembled without regard to cross-species conservation. The co-expression* set was identified by taking the top *n* most-co-expressed genes, where *n* was the total number of targets identified by ARACNe rather than based on a predefined p-value threshold (see Co-expression in **Materials and methods**). Note that there is no statistical threshold that could be used to reproduce the same selection a priori. Hence the co-expression* set can only be defined once ARACNe has been run and it was used only to determine if ARACNe further improves over co-expression even if only the most co-expressed targets are considered.

For each TF, the single most enriched motif was compared to the TRANSFAC reported motifs for that TF. Success was reported if a match was found using matcompare [35]. The recall rate for conservation-free, co-expression, and co-expression*, was 27/70 (39%), 13/70 (19%), and 25/70 (36%), respectively (Table 1). In our experiments, ARACNe (conservation-free set) significantly outperformed co-expression (p<0.05, by FET), and more narrowly outperformed co-expression*. This result suggests that ARACNe significantly improves over co-expression approaches by removing some false positives among high-scoring co-expressed targets and many false positives among low-confidence co-expressed targets. Overall, the conservation-free set consistently had the highest recall rate, and its inferred targets were used for all subsequent experiments. Note that selection of the single most enriched motif for estimating recall is an exceedingly strict criterion. Indeed, we show that ubiquitous co-factors may in fact be more enriched than the TF-specific binding motif itself. As a result, the correct motif can be recovered for much more than 39% of the TFs if additional, statistically significant motifs, are also considered. For instance, when the criteria for correct identification was expanded to include the top 5 motifs (see Motif evaluation and discovery in **Materials and methods** and Table S5), recall improved to 48/70 (68%).

**Table 1:** Motif predictions comparison.

	total TFs	True Positives (matched TFs)	Recall
**ARACNe**	70	27	38.57%
**Coexpression**	70	13	18.57%
Coexpression*	70	25	35.71%
**ARACNe-MRC**	70	25	35.71%

We compared motif enrichment accuracy across promoters corresponding to targets identified by the regulatory-network reverse-engineering algorithm ARACNe, co-expression, and a combination of both methods (see Results). ARACNe-MRC corresponding to ARACNe inferred target promoters with exons and repeats masked; see Table S1 for expanded description.

### Cross-species conservation analysis further improves TFBS discovery

Many functional elements, including TFBSs, are conserved across species [18], [19], but the proportion of TFBS conservation that can be identified directly from genome alignments is still unknown. Ward and Bussemaker [22] and Xie et al. [29] suggested using both alignment-based and alignment-independent approaches to identify evolutionary conserved regions. We studied the benefit of cross-species conservation in ARACNe-identified promoters for 70 representative TFs. Analysis was performed by sequence alignment, by pattern discovery using SPLASH [10], [36], and by a combination of the two methods. Since pattern discovery is especially sensitive to the presence of repeats and large highly-conserved regions, we first masked out repeats and coding exons. We processed the sequence data using the same procedure as described above to assess the recall rate for the known TFBS of the 70 representative TFs.

**Figure 1:**
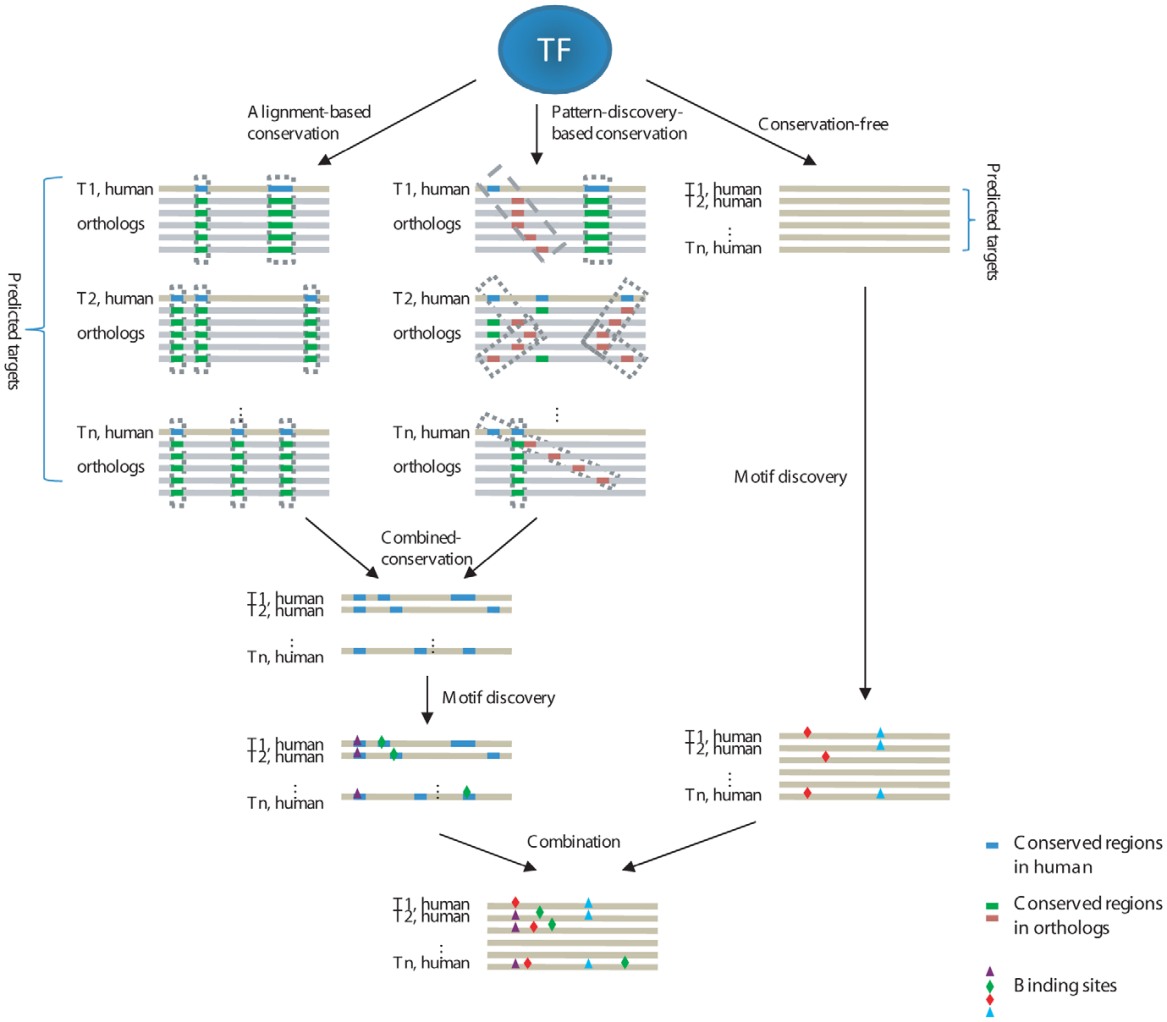
OmniMiner's motif discovery workflow. For each TF, we identified target genes for the TFs and assembled a set of promoters corresponding to these genes. Cross-species conserved regions were identified in these promoters using alignment-based and pattern-discovery-based methods and were combined to produce the combined-conservation set. Motif discovery was performed separately on the original promoters and the combined-conservation set. The resulting motifs were merged and re-ranked according to their classification relative-error rate.

After masking repeats and coding exons, but before conservation analysis, the recall rate was slightly reduced, from 27/70 (39%) to 25/70 (36%), due to loss of some bona-fide TFBSs in masked regions (see Tables 1 and S1). However, this loss is requires for conservation analysis and is justified by the benefit of cross-species conservation. Additionally, the affected motifs for the two TFs were still ranked in the top five (see Table S1). In order to study the benefit of alignment-based conservation analysis, we used phastCons [27] to identify the most conserved sequence fraction that would optimize recall (see Table 2), where this fraction is defined as the percent of nucleic acids in the sequences retained after masking poorly conserved regions. Surprisingly, the optimal recall rate using alignment-based conservation was only 25/70 (36%) at 10% DNA coverage, showing no improvement. We supplemented the alignment-based cross-species conservation with pattern-discovery-based (alignment-free) analysis. Specifically, we first identified conserved patterns in each masked orthologous promoter set with SPLASH. We then selected the sequence fragments covered by the most statistically significant SPLASH patterns until the desired DNA coverage was achieved. This was also set to 10% to ensure results that are comparable with alignment-based conservation analysis. We refer to the resulting promoter fragment sets produced by the combination of phastCons and SPLASH analysis as the *combined-conservation set*. Analysis of the combined-conservation set improved the prediction recall rate to 31/70 (44%). Finally, we merged motif enrichment results independently produced by the conservation-free and the combined-conservation sets, re-ranking motifs according to the best classification relative-error rate achieved in either test (see Figure 1). The resulting recall rate increased further to 35/70 (50%). Thus, use of cross-species conservation data, within an integrative framework significantly (p<0.05, by FET) improved recall rate, and joint use of alignment- and pattern-discovery-based approaches yielded an additional statistically significantly improvement (p<0.05, by FET) over either method in isolation.

**Table 2:** Motif predictions based on conservation-free and conserved promoters.

	total TFs	True Positives (matched TFs)	Recall
**Conservation-free**	70	27	38.57%
**Alignment-based Conservation**			
** 5%**	70	7	10%
** 10%**	70	25	35.71%
** 20%**	70	22	31.43%
** 25%**	70	18	25.71%
**Combined-conservation**			
** 10%**	70	31	44.29%
**Conservation-free and Combined-conservation merged**	70	38	54.29%

All promoters used in these predictions were inferred by ARACNe algorithm. We compared motif enrichment accuracy across the original promoters and conserved regions identified using alignment-based conservation with varying DNA-coverage proportions, and a combination of alignment-based and pattern-discovery based conservation. For alignment-based conservation, best performance was achieved at 10% DNA coverage, and this was used in conjunction with pattern-discovery based conservation at 10% DNA coverage to produce combined-conservation. A test is considered as successful if the most enriched motif identified using either the conservation-free or the combined-conservation promoters matched the known motif for the TF. We called it conservation-free and combined-conservation merged. The recall rate at this level was significantly better than that using conservation-free alone.

### Testing *de novo* motif discovery

**Figure 2:**
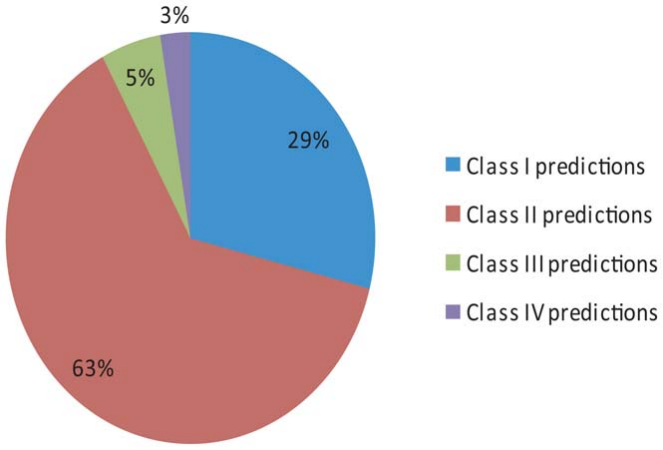
*De novo* motif-discovery accuracy measurement. *De novo* motif-discovery was performed on the 38 TFs for which the known TFBSs were enriched in the target genes. Predicted motifs were classified into four classes. Class I: the top three predictions included a significant classifying motif than matched the known motif for the TF. Class II: a lower-ranking significant classifying motif that matched the known motif for the TF. Class III: The most enriched motif was a significant classifier, but no motifs matching the known motif for the TF. Class IV: no significant classifiers were found.

To estimate OmniMiner's accuracy and determine if our test set has is rich enough for *de novo* motif discovery, we tested whether TFBSs for the 38 TF, whose TRANSFAC motifs were correctly identified in the previous subsection (on either conservation-free or combined-conservation sets) could also be identified *de novo*. We also used the analysis of the combined-conservation set to compare the performance of OmniMiner to that of GibbsModule. Specifically, we ran DME [11] on both the conservation-free and the combined-conservation *sets* and recorded p-values for DME-identified motifs, reporting motifs with p<0.05 (see Motif evaluation and discovery in **Materials and methods**). Following the same procedure described for TRANSFAC motifs, we re-ranked significant motifs based on the best classification relative error rate achieved on either the conservation-free or the combined-conservation sets. We considered DME to be successful if a top *de novo* discovered motif matched a known motif for the TF according to matcompare. Results are given in Figure 2. For 32/38 (84%) of the TFs, we were able to discover significantly enriched motifs that matched the reported TF motif in TRANSFAC. Strictly matching significant motifs among the top 5 motifs per TF were recovered for 2/38, 13/38 and 10/38 of the TFs on the conservation-free, combined-conservation, and the combination of the two, respectively. The result suggests that, likely because of their length and count, cross-species conservation is required for *de novo* discovery on our promoter test sets.

In order to compare OmniMiner on combined-conservation promoters to GibbsModule, we ran GibbsModule on the conservation-free *set* with the orthologous promoters as additional input. GibbsModule performs cross-species conservation analysis internally, but it does not output p-value information or motif ranking. In the absence of ranking, we used all GibbsModule-discovered motifs, and for fairness, compared the 9 GibbModule-discovered motifs both to the top 3 and to the top 9 DME-discovered motifs with no p-value restriction. For 12/38 (31%) of the TFs, one of the nine GibbsModule-discovered motifs matched a known motif for the TF. This performance is significantly worse than DME's recall rate of 21/38 (55%) when considering the top nine ranking motifs, and it is also worse than the recall rate of 14/38 (37%) when only the top three DME motifs are considered (see Table 3).

**Table 3:** Performance comparison of OmniMiner to GibbsModule.

	total TFs	True Positives (matched TFs)	Recall
**DME-Total**			
** (top 3)**	38	11	28.95%
** (top 3)** *	38	14	36.84%
** (top 9)**	38	17	44.74%
** (top 9)** *	38	21	55.26%
**GibbsModule**			
** (best 9)**	38	12	31.58%

We compared OmniMiner and GibbsModule recalls on our 38 TFs test set.

DME-Total used both the conservation-free and the combined-conservation promoter sets.

*No p-value threshold was used for pruning motifs.

To test DME-discovered motifs on an external data source, we tested for site enrichment for the top three predicted ZNF263 motifs in the top 500 ZNF263-bound regions according to ChIP-seq in K562 cells relative to unbound regions after size correction. The results show that all top motifs are highly enriched in ZNF263-bound regions with respective p-values of 2.35e-57, 3.80e-19 and 9.01e-3 (see Validation in **Materials and methods**).

### Test set clusters

**Figure 3:**
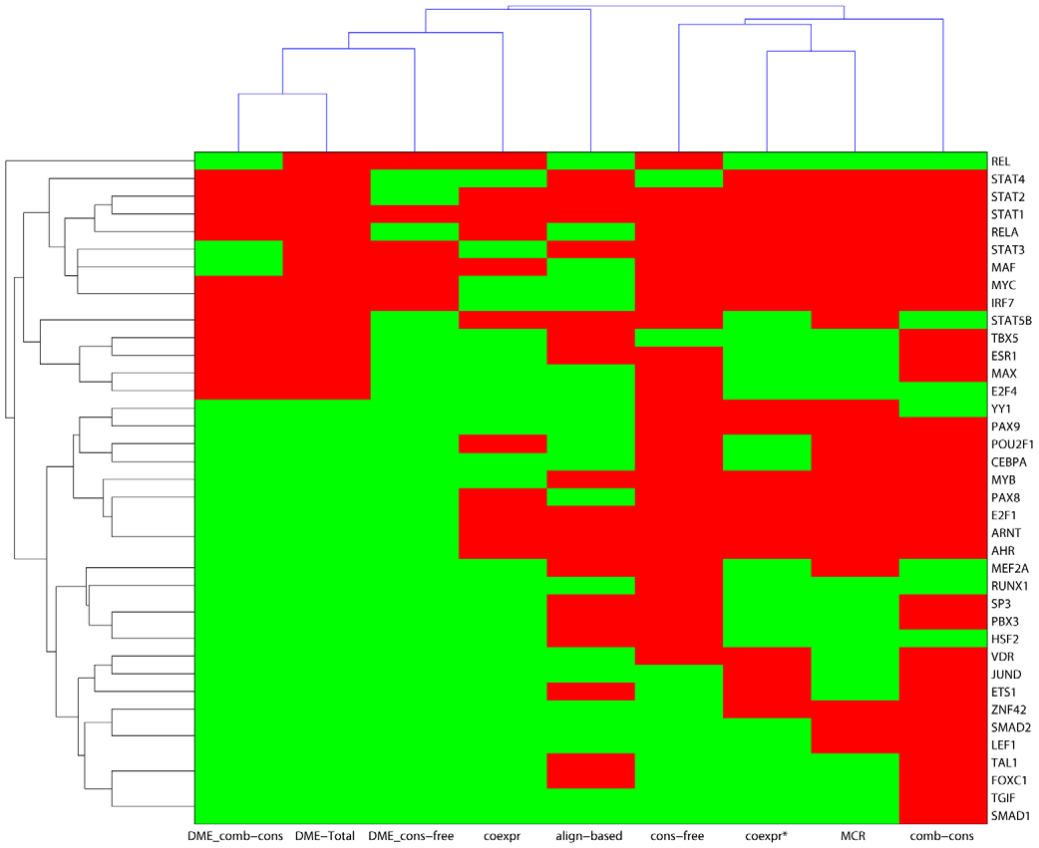
Classification of motif prediction. We classified the 38 TFs used as the test set and the motif discovery methods according to enrichment and discovery success. DME_comb-cons stands for the comb-cons promoter set that was used for *de novo* motif discovery. DME-Total represents the result from combining *de novo* motifs discovered in the conservation-free and combined-conservation sets. DME_cons-free stands for the cons-free promoter set that were used for *de novo* motif discovery. Coexpr represents the promoter set inferred by Spearman correlation. Align-based represents the promoter set in which the conservations were identified by alignment-based method. Cons-free represents the conservation-free set. Coexpr* represents the promoter set inferred by the combination of the ARACNe and coexpression. MCR represent the conservation-free set with exons and repeats masked. Comb-cons stands for the promoter set in which the conservations were identified either by a combination of alignment-based and pattern-discovery-based methods.

We clustered test sets and motif discovery methods according to motif discovery success; see Figure 3. The clustering results show clear TF grouping according to binding site identification and discovery methods, suggesting that, for some TFs, binding site enrichment can be detected using most methods. However, for some TFs, TFBS enrichment is only detectable when cross-species conservation data is used. For example, STAT1, STAT2, STAT4, STAT3, RELA, MAF, MYC and IRF7, which form one cluster, were correctly classified with and without conservation analysis, and using known motifs or *de novo* discovered motifs. Members of a second cluster, including PAX9, POU2F1, CEBPA, MYB, PAX8, E2F1, ARNT, and AHR1, were correctly classified with and without conservation but not using *de novo* motif discovery. Finally, members of a third cluster, including JUND, ETS1, ZNF42, SMAD2, LEF1, TAL1, FOXC1, TGIF, and SMAD1, were correctly classified with the help of cross-species conservation but not in the original conservation-free promoter sets.

### New predictions and biochemical validation

**Figure 4:**
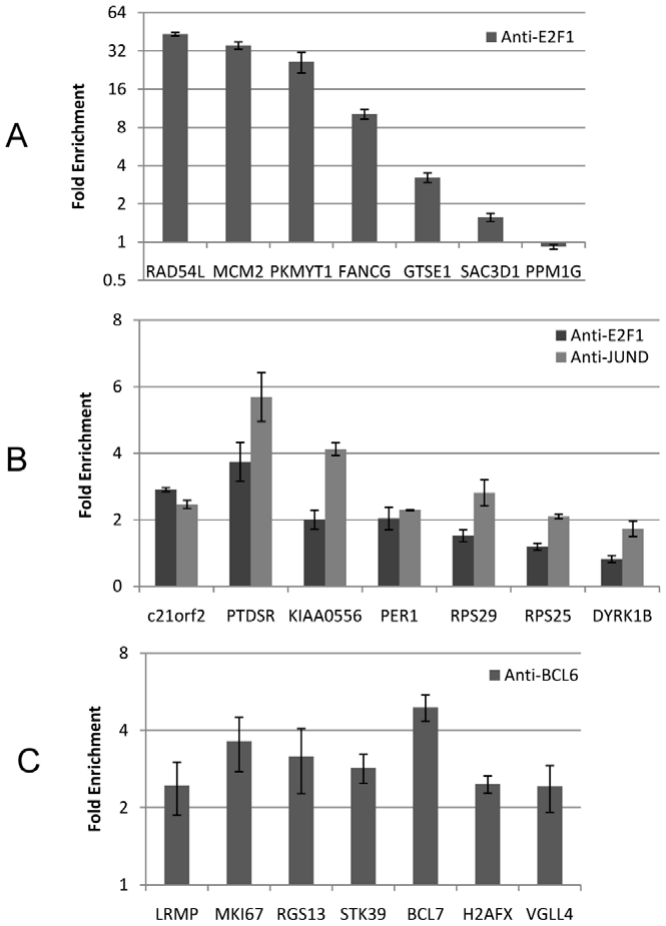
Binding validation. (**A**) E2F1 binding to predicted E2F1 targets. (**B**) E2F1 and JUND binding to predicted JUND targets. (**C**) BCL6 binding to predicted BCL6 target. We plot fold enrichment relative to IgG (mean ± s.e.m).

The TRANSFAC E2F1 DNA-binding motif M00918 was the most enriched motif with sites in promoters of predicted E2F1 targets. As proof of principle, we tested top-scoring sites for M00918 in seven randomly-selected promoters using quantitative PCR of chromatin immunoprecipitation assays (qChIP). Our results show that E2F1 binds to top predicted sites in the promoters of RAD54L, MCM2, PKMYT1, FANCG and GTSE1. We failed to show binding to top sites in the promoters of SAC3D1 and PPM1G (Figure 4**A**).

No motif was significantly enriched in the JUND conservation-free *promoter sets*. However, both JUN and E2F-1 motifs (M00428 and M000172) were among the top 5 motifs for all JUND test sets, and they were significantly enriched in the JUND combined-conservation set, where the reported AP1 and E2F1 motifs were the most enriched motifs. Consistent with these predictions, we show that both E2F1 and JUND bind to the best matching sites in the promoters of predicted targets C21orf2, PTDSR, KIAA0556 and PER1, suggesting that this transcriptional co-binding pattern may be pervasively used in human B cells. However, while we were able to show that JUND binds to RPS29 and RPS25, we could not detect enrichment of E2F1 antibody by qChIP at the top candidate sites in their promoters. Similarly, we failed to show enrichment of either TF's antibody to the promoter of the predicted target DYRK1B (Figure 4**B**). This suggests that either the qChIP analysis produced a false negative result or that these sites were false positive predictions. In total, we validated the top predicted sites for JUND binding in 6/7 of the predicted JUND targets, and we validated E2F1 binding sites in 4/6 of the JUND bound promoters.

Finally, we proceeded to predict *de novo* DNA-binding motifs for twenty TFs with previously uncharacterized binding motifs. The top three predicted motifs with p-value<0.05 (see Motif evaluation and discovery in **Materials and methods**) for each TF are given in Table S2; only two significant motifs were identified for NME2 and EP300. Experiments on our test set suggest that *de novo* motif discovery is able to identify significant binding motifs for the vast majority of TFs. Because of antibody availability, we chose to validate binding sites for the top BCL6-predicted motif. Our results show that BCL6 binds to the promoters of LRMP, MKI67, RGS13, STK39, BCL7, H2AFX and VGLL4 (Figure 4**C**). Indeed, all tested promoters were validated for BCL6 binding. The BCL6 motif was identified from the BCL6 combined-conservation set, and matched a previously reported BCL6 half site [37].

## Discussion

Here, we proposed a novel integrative methodology that combines reverse-engineering of transcriptional networks and cross-species conservation analysis for TF binding-motif discovery. We produced *de novo* motif predictions for 20 previously uncharacterized TFs, and validated site predictions for the top BCL6 motif and for co-binding patterns between E2F1 and JUND. In order to compare methods, we produced an extensive test set of co-regulated human genes and promoters in B cells; these test sets are given in Table S3. Our results suggest that 50% of the transcriptional regulators analyzed by ARACNe in a human B cell context produce inferred target sets that are significantly enriched in their bona-fide TF functional direct targets. This is a lower-bound for the proportion of TFs for which bona-fide targets can be identified since (a) only a relatively small region of the promoter was considered, (b) some TFs are poorly characterized in TRANSFAC, (c) only predicted activated targets were considered, (d) some TFBS motifs are highly degenerate and may not be reconstructed from enrichment analysis alone, and (e) we defined success restrictively, requiring the top predicted motif to match a known motif to the TF and disregarding the possibility of a match to a co-factor motif.

The novelty in our approach was three-fold. First, we showed that using a reverse-engineering algorithm instead of gene co-expression to identify TFBS-enriched promoter sets significantly improved prediction. Second, we showed that using a combination of alignment- and pattern-discovery-based conservation analysis approaches significantly improves prediction when compared to using only one of the approaches. Third, we showed that by combining the two approaches, we can further improve prediction accuracy and almost doubled the 12/38 (31%) recall of another recent integrative approach (GibbsModule). Finally, we produced predictions for 20 TFs with previously unknown binding affinity and validated predictions by quantitative Chromatin Immunoprecipitation assays (qChIP) and enrichment in ChIP-seq data. By developing a unique test set of human promoters and conserved regulatory regions, we were able to produce realistic estimates for the quality of our *de novo* prediction method.

We used stringent criteria to test our input sets, requiring that the most enriched TRANSFAC motif in a foreground set be similar to a known motif for the TF. Based on this metric, even before cross-species conservation is used, nearly 40% of the tested TF motifs pass this criterion. This is a significantly higher recall rate than the one observed when using co-expression. To understand the source of this performance gap, we compared the two methods to a hybrid method. Instead of using a p-value cutoff for co-expression, we used the number of ARACNe predicted activated targets as a cutoff. The hybrid method performed only marginally worse than ARACNe, suggesting that genes with expression profiles that are most similar to the TF's are the most likely targets and that ARACNe's main advantage is in a highly TF-dependent and accurate co-expression similarity cutoff selection.

Maybe the most surprising result in this study was that using alignment-based conservation to identify regions enriched with TFBSs did not improve recall. This suggests that removing less conserved regions, in an effort to reduce background noise, may lead to loss of regions containing bona-fide TF biding sites. On the other hand, using non-linear pattern-discovery-based conservation improved the performance considerably and use of both methods in combination provided the best results. Cross-species conservation significantly improved recall, but only when jointly considering sequence fragments discovered both with alignment-based and pattern-discovery-based analysis. For seven TFs, known motifs were found to be the most enriched only when using the entire promoter sequence, suggesting that evolution of their transcriptional targets may be more recent and poorly conserved in orthologous species or that our alignment techniques are not sensitive enough for this task. Our conservation-free promoter sets appear to be either too long or too few for *de novo* discovery, which was successful only on the combined-conservation set, and for 18/20 of the TFs for which we discovered *de novo* DNA-binding motifs, including the validated BCL6, the top motifs were selected from the combined-conservation set analysis.

Figure 3 suggests that our test sets can be clustered according to motif discovery success, with one 8-test-set cluster consisting of promoters that were correctly classified without conservation, with the aid of alignment-free conservation, and by *de novo* motif discovery. However, only 4/8 of the sets were correctly classified using alignment-based cross-species conservation. Our findings support the idea that TFBS conservation is fundamentally different from coding-region conservation. This may be due to operating distance flexibility, *cis*-regulatory module grammar, or neutral mutations in site positions that correspond to low-information motif columns.

Despite these significant advances, we could not identify known TFBS motifs for several of the TFs, suggesting that these are either poorly characterized in TRANSFAC, that binding for that promoter is supported by heterogeneous mechanisms, or that reverse-engineering may fail to appropriately characterize the transcriptional targets of some TFs. This, in turn, affirms that the problem of TF binding-site characterization is still open and much remains unknown. It also suggests a set of TFs that may be especially hard to characterize. An important point is that our ability to characterize TF binding motifs is likely cell-context dependent. We used a large gene expression profile dataset for mature human B cells, which may have both improved our ability to characterized some TFs as well as hinder the ability to characterize others. Analyses of similar datasets from other cellular contexts may help answer these questions.

Machine learning heuristics fall in one of three categories: heuristics that search for good solutions in complete problem domains but do not guarantee optimality, heuristics that discover the best solutions in simplified problem domains, and those that search in simplified problem domains but do not guarantee optimality. GibbsModule arguably falls in the first category, while DME, SPLASH and OmniMiner belong to the second category. We previously showed that DME outperforms other motif discovery algorithms on both synthetic and mammalian data. The argument in favor of DME [14] is based on properties of the motif-discovery solution-space structure, which under a variety of formulations is in 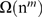 and 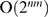, where *m* denotes the number of input sequences and *n* denotes their length. This space is smooth and allows for local optima discovery, making DME's fine grid search followed by a locally optimal refinement a successful strategy. In the presence of orthologous promoters, the search space is in Ω(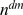), where *d* is the number of ortholog species used. Moreover, there is no proven formulation for the integration of the two orthogonal optimization criteria. We hypothesize that due to the computationally prohibitive task of identifying patterns across sequences with varying degrees of similarity and in the absence of a demonstrably good type-1 method, a type-2 heuristic should be preferred. Finally, our success in identifying pattern-discovery-based conserved regions is due to SPLASH's ability to identify long and sparsely conserved regions. Thus, SPALSH is able to overcome some of the limitations of linear multiple-sequence aligners, and specifically it does not discard sites due to varying module grammar, or neutral mutations. We followed SPLASH conserved-region identification with motif discovery by DME to identify conserved motifs in these regions, thus fixing motif column values whether they have high or low information content.

To create a realistic testing platform for motif discovery in human regulatory regions, we identified promoter sets that were predicted to be co-regulated by known TFs, and are significantly enriched with a motif associated with these TF. This platform allowed us to estimate the accuracy of our motif discovery methods. The platform is composed of 38 human promoter sets of varying sizes and it is computationally validated. Its size, validation, and specialization make it a unique platform for motif discovery evaluation. Our tests with *de novo* motif discovery suggest that we recover 12/38 of the known motifs associated with the query TF, and we identify significant motifs for 36/38 (p<0.01) of these TFs (see Table S1).

## Materials and Methods

### ARACNe Network Inference

ARACNe is an information-theoretic method for identifying transcriptional interactions between TFs and their targets using gene expression profile (GEP) data. In brief, the algorithm first distinguishes candidate interactions between a TF and its targets by estimating the expression pairwise mutual information (MI). Interactions with significant MI values are retained (details can be found at ref 14). Then, ARACNe applies the Data Processing Inequality (DPI) theorem to eliminate the vast majority of interactions with significant MI values that are indirect and falsely predicted because of transcriptional interaction cascades. ARACNe with bootstraps uses bootstrap sampling during network reconstruction to non-parametrically assess statistical confidence for predicted transcriptional interactions. As a result, the built networks are more robust to both expression estimation and MI estimation errors. Dataset samples were randomly chosen with replacement and assembled into bootstrap datasets. In our experiments, 100 bootstrap datasets were generated and ARACNe was used to generate a set of bootstrap networks. Each bootstrap network contributed to a consensus network made of edges that were supported across a significant number of the bootstrap networks, where significance was measured using permutation testing with the null generated using shuffled networks and cutoff set to p<1e-7 to correct for multiple testing.

### Co-expression

We used 254 gene-expression profiles collected from a variety of homogeneous B cell phenotypes by Basso et al. (2005) using the Affymetrix HG-U95A GeneChip® System; experimentally manipulated cell lines were excluded. TFs were selected among the genes represented on the HG-U95A microarray based on Gene Ontology annotation (Table S3).

For each TF we identified (a) a set of co-expressed genes using Spearman correlation with a Bonferroni-corrected statistical threshold of 1e-4, and (b) a set of ARACNe inferred targets using a Mutual-Information-based Bonferroni-corrected significance threshold of 5e-2 and recorded positively correlated targets with each regulator. The Spearman correlation threshold was set low because higher threshold settings produced significantly larger target-gene sets and poor analysis results. ARACNe predicted TF target sets of size 30 or greater for seventy TFs with verified binding motifs in TRANSFAC. These TFs were used to compile our test set. The number of statistically co-expressed genes with each TF was significantly larger than the number of ARACNe-identified targets, and analysis results showed that ARACNe-identified targets are significantly more enriched with sites matching validated binding site motifs. To test if the disparity in site enrichment was related to the disparity in target set sizes, we selected the top *n* most statistically significant co-expressed targets for each TF, where *n* was the size of the ARACNe target set for this TF. This test set makes the co-expression* set; see Table S3 for target set identities according to the three methods.

### TF target sequences

We obtained 1500 bp promoters for each target gene by selecting [−1000, 500] from Refseq transcription start site locations, eliminating intersecting promoters arbitrarily; we refer to these as the *conservation-free* sets. We masked repeats and coding exons to obtain the Masked Coding-exons and Repeats (*MCR*) set, which was used for computing pattern-discovery-based cross-species conservation. Alignment-based conservation was computed using 17-species PhastCons [27]. PhastCons requires a conservation probability parameter; we mapped the conservation probability parameter to DNA coverage proportion in order to achieve comparable statistics across regulators and conservation measures. A mapping of conservation probability to DNA coverage proportion for the seventy TFs is given in Figure S1. To add pattern-discovery-based conservation, we retrieved orthologous promoters for mouse, rat, chimpanzee, rhesus and dog. We used SPLASH [10], [36], a deterministic pattern discovery algorithm, to identify patterns across species after masking repeats and coding exons. When running SPLASH, we used eight-base windows for motif-seed discovery with a minimum six-base match within the window, and required a match across at least four species. SPLASH-identified conserved patterns were ranked by z-scores, and the top patterns were used to achieve a given DNA coverage proportion. Entire regions included in a sparse pattern were considered conserved. We combined alignment-based conservations at 10% DNA coverage with pattern-discovery-based conservations at 10% coverage to construct combined conservation target sequences (conservation*). Regions that were not considered conserved according to pattern-discovery-based or alignment-based conservation were masked out.

Our control set (background) was composed of 2000 non-overlapping promoters associated with randomly selected Refseq genes not identified as ARACNe or co-expression targets. These promoters were processed to obtain a background MCR set, alignment-free regions, and combined conservation sequences. When evaluating or discovering motifs enriched in a foreground set, we used the background set whose processing matched the processing of the foreground set.

### Motif evaluation and discovery

*De novo* motif discovery was performed for TFs with significant binding site enrichment and for 20 TFs with no known binding characterization. We identified 103 TFs that activated at least 30 targets and had no known associated motifs. We ranked these TFs based on the number of PubMed abstracts containing the name of the TF (Table S4). *De novo* motif discovery was performed for the top 20 most cited TFs.

Motif enrichment in foreground sets against background sets was measured using classification relative error rate (*err*), where relative error rate is computed as the average of the false positive and false negative rates [11]. Relative error rates were associated with p-values using permutation testing, where the indicator vector that assigns set membership to foreground or background is randomly permuted. When identifying discriminating motifs in a motif library, we assigned a p-value to an error rate by ranking it relative to the library's top error rates in 10,000 permutation tests. When assigning p-values to *de novo* identified motifs, we first generated 100 random foreground-background pair sets by permuting the indicator vector as described above. We then applied DME [14] to each of the 100 random foreground-background pair sets. In each permutation test, the score of the motif with the lowest relative error was recorded, and the resulting set of 100 relative error rates served as a null distribution against which we assessed the statistical significance of the *de novo* identified motifs from the original set. Motifs in the 95^th^ percentile (p≤0.05) are said to be statistically significant.

We used *matcompare* [35] with a similarity cutoff of 1.0 bit for motif comparison. DME [14] was used to discover enriched motifs of length 6, 8, and 10. Similar top motifs were merged using uniqmotifs [11]. *GibbsModule* [29] was used to identify motifs of length 8, 10 and 12 with the default 300 iteration per execution.

### Validation

We set out to validate ARACNe target predictions, TRNASFAC-based binding site predictions, co-factor binding predictions, and *de novo* motif discovery predictions. With consideration to anti-body availability, we chose to validate binding predictions for three TFs. The TRASNFAC E2F1 motif M00918 was identified as the most enriched motif in E2F1 targets, and the TRASNFAC E2F1 motif M00428 was identified as the most enriched motif in JUND targets. We validated BCL6 binding to sites identified using the top BCL6 motif candidate. Antibodies used for the study were anti-E2F1 (sc-251), anti-JUND (sc-74), anti-BCL6 (sc-585) and anti-GAPDH (sc-32233) from Santa Cruz Biotechnology.

Chromatin immunoprecipitation (ChIP) analysis was done in Ramos and MUTU-I cell lines by following the protocol described by [38]. Ramos and MUTU-I cells were maintained in Iscove's modified Dulbecco's medium supplemented with 10% FBS and antibiotics. The soluble chromatin fraction was immunoprecipitated with anti-E2F1 or mouse IgG control antibody (MUTU-I), anti-JUND or rabbit IgG control antibody (MUTU-I), and anti-BCL6 or mouse IgG control antibody (Ramos). The immunoprecipitated DNA was reverse cross-linked and purified by phenol-chloroform. The chromatin fragments from two independent experiments were pooled and the amount of DNA immunoprecipitated by an individual antibody was assessed by real-time PCR in 7300 Real-time PCR System using Power SYBR Green (Applied Biosystems).

Two ZNF263ChIP-seq replicate experiments and IgG control in K562 cell line were obtained from UCSD ENCODE Data Release: Transcription Factor Binding Sites from Yale/UC-Davis/Harvard. MACS [39] was used under default settings to predict (1) ZNF263 and (2) IgG bound regions. We used the top 500 ZNF263-bound regions as foreground, and as background we selected the top IgG bound regions to equal the total DNA of the foreground. Motif training and binding site detection followed the process described above, and enrichment p-values were calculated using Fisher exact test, comparing detection rates in each set, with Bonferroni correction for multiple testing.

## Supporting Information

Figure S1 (0.05 MB PDF)

Table S1 (0.06 MB XLS)

Table S2 (0.03 MB PDF)

Table S3 (8.49 MB ZIP)

Table S4 (0.02 MB XLS)

Table S5 (0.03 MB XLS)
