# Supplementary material for: A Systems Biology Approach to Transcription Factor Binding Site Prediction
Source: PLoS One. 2010 Mar 26;5(3):e9878. doi: 10.1371/journal.pone.0009878 (PMC2845628; doi:10.1371/journal.pone.0009878)
Supplement: Figure S1 — (0.05 MB PDF) [file pone.0009878.s001.pdf]

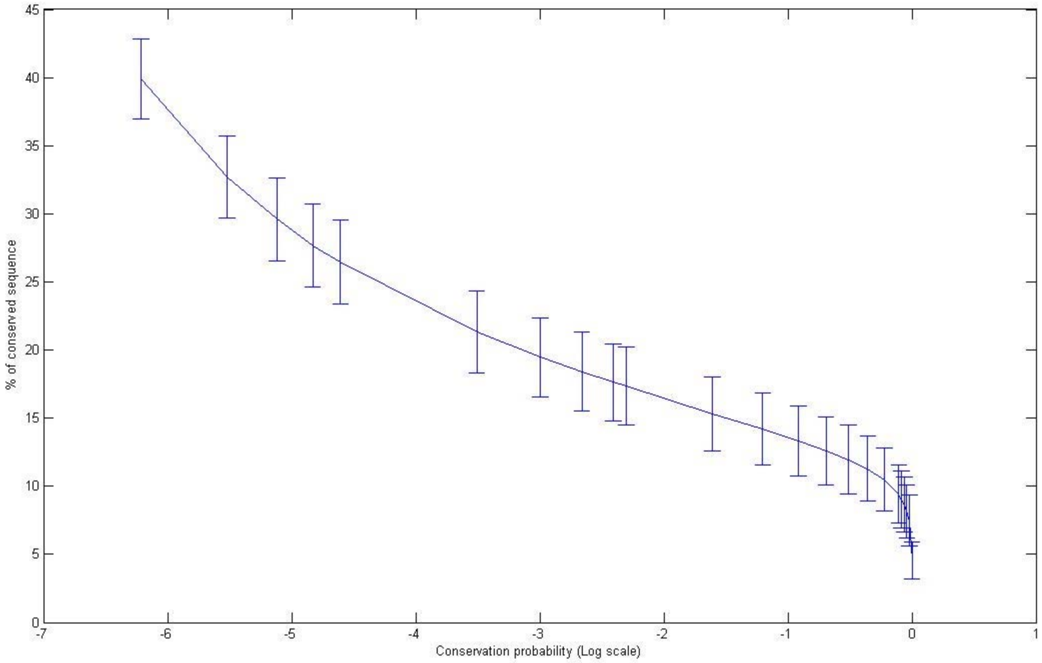

Supplementary Figure 1. phastCons conservation probabilities and corresponding conserved-sequence proportions.
