# Supplementary material for: A Systems Biology Approach to Transcription Factor Binding Site Prediction
Source: PLoS One. 2010 Mar 26;5(3):e9878. doi: 10.1371/journal.pone.0009878 (PMC2845628; doi:10.1371/journal.pone.0009878)
Supplement: Table S2 — (0.03 MB PDF) [file pone.0009878.s003.pdf]

TF	Source	Err	Sens	Spec	p-val	Logo
BCL6	Cons	0.29	0.73	0.68	0.00	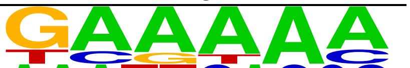
BCL6	Cons	0.30	0.80	0.60	0.00	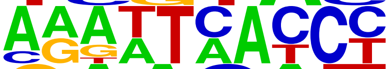
BCL6	Cons	0.30	0.80	0.59	0.00	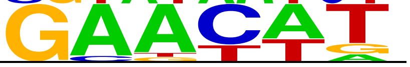

TF	Source	Err	Sens	Spec	p-val	Logo
NFATC1	Cons	0.27	0.78	0.69	0.00	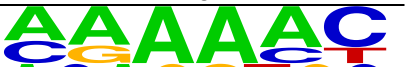
NFATC1	Cons	0.27	0.75	0.71	0.00	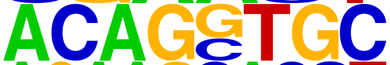
NFATC1	Cons	0.27	0.72	0.73	0.00	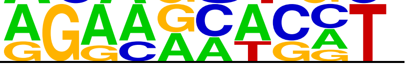

TF	Source	Err	Sens	Spec	p-val	Logo
NME2	Cons	0.28	0.72	0.72	0.00	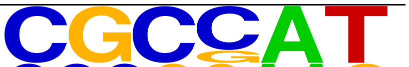
NME2	Cons	0.30	0.73	0.68	0.00	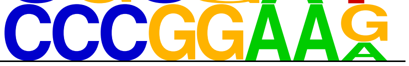

TF	Source	Err	Sens	Spec	p-val	Logo
RB1	Cons	0.26	0.92	0.57	0.00	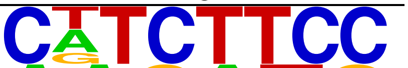
RB1	Cons	0.28	0.70	0.73	0.00	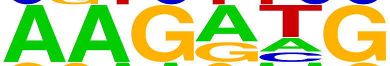
RB1	Cons	0.28	0.73	0.70	0.00	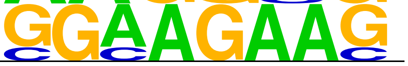

TF	Source	Err	Sens	Spec	p-val	Logo
VAV1	Cons	0.24	0.81	0.71	0.00	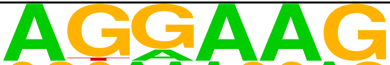
VAV1	Cons	0.24	0.76	0.76	0.00	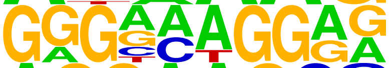
VAV1	Cons	0.25	0.78	0.73	0.00	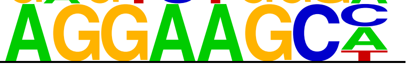

TF	Source	Err	Sens	Spec	p-val	Logo
NFE2L2	Cons	0.28	0.91	0.54	0.00	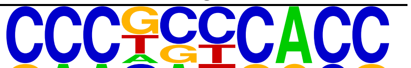
NFE2L2	Cons	0.28	0.70	0.74	0.00	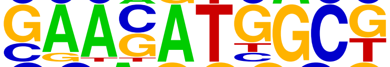
NFE2L2	Cons	0.29	0.97	0.45	0.00	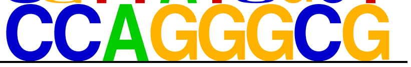

TF	Source	Err	Sens	Spec	p-val	Logo
MECP2	Cons	0.23	0.85	0.68	0.00	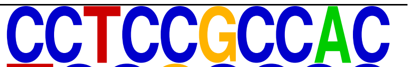
MECP2	Cons	0.25	0.96	0.55	0.00	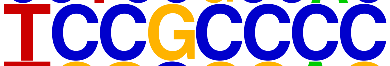
MECP2	Cons	0.25	0.70	0.80	0.00	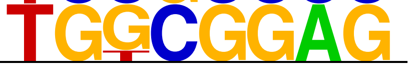

TF	Source	Err	Sens	Spec	p-val	Logo
FLI1	Orig	0.28	0.69	0.75	0.00	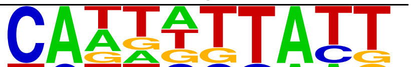
FLI1	Orig	0.30	0.66	0.75	0.00	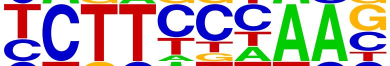
FLI1	Orig	0.30	0.77	0.64	0.00	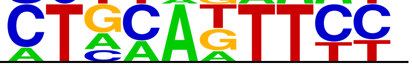

TF	Source	Err	Sens	Spec	p-val	Logo
HOXD13	Cons	0.32	0.73	0.63	0.00	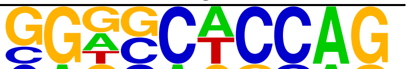
HOXD13	Cons	0.32	0.73	0.63	0.00	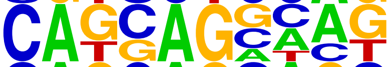
HOXD13	Cons	0.32	0.60	0.75	0.00	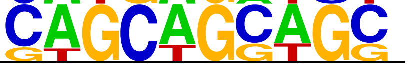

TF	Source	Err	Sens	Spec	p-val	Logo
HIF1A	Cons	0.27	0.71	0.76	0.00	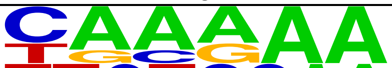
HIF1A	Cons	0.30	0.83	0.57	0.00	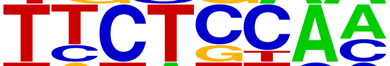
HIF1A	Cons	0.30	0.80	0.59	0.00	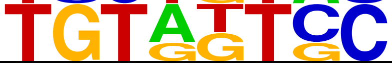

TF	Source	Err	Sens	Spec	p-val	Logo
ID1	Cons	0.31	0.83	0.54	0.00	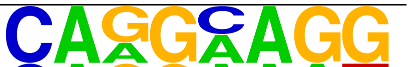
ID1	Cons	0.32	0.68	0.69	0.00	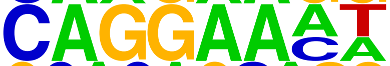
ID1	Orig	0.33	0.58	0.77	0.00	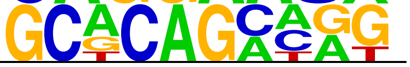

TF	Source	Err	Sens	Spec	p-val	Logo
MEF2C	Orig	0.29	0.77	0.64	0.00	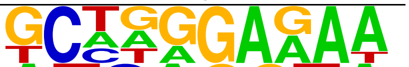
MEF2C	Cons	0.31	0.77	0.61	0.00	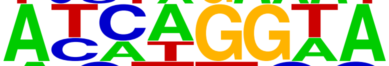
MEF2C	Cons	0.32	0.69	0.67	0.00	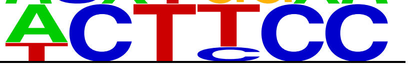

TF	Source	Err	Sens	Spec	p-val	Logo
PAX7	Cons	0.32	0.71	0.65	0.00	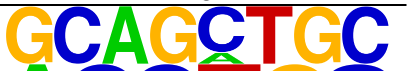
PAX7	Cons	0.33	0.72	0.62	0.00	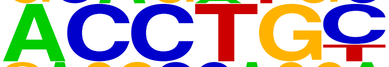
PAX7	Orig	0.33	0.75	0.60	0.05	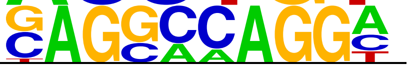

TF	Source	Err	Sens	Spec	p-val	Logo
TBX1	Cons	0.25	0.74	0.76	0.00	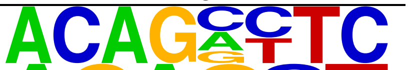
TBX1	Cons	0.25	0.77	0.72	0.00	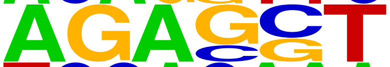
TBX1	Cons	0.27	0.74	0.72	0.00	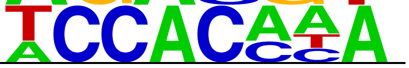

TF	Source	Err	Sens	Spec	p-val	Logo
MLL	Cons	0.32	0.76	0.60	0.00	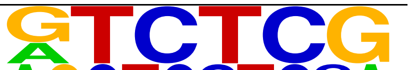
MLL	Cons	0.32	0.75	0.61	0.00	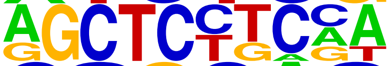
MLL	Cons	0.33	0.88	0.47	0.00	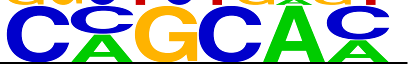

TF	Source	Err	Sens	Spec	p-val	Logo
APC	Cons	0.26	0.78	0.71	0.00	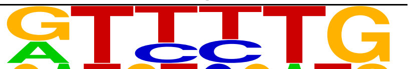
APC	Cons	0.27	0.74	0.71	0.01	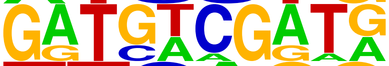
APC	Cons	0.28	0.63	0.81	0.02	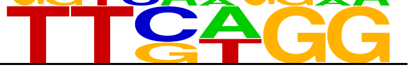

TF	Source	Err	Sens	Spec	p-val	Logo
CEBPZ	Cons	0.27	0.72	0.75	0.00	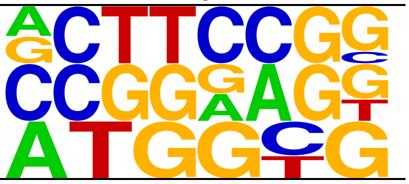
CEBPZ	Cons	0.27	0.84	0.61	0.00	
CEBPZ	Cons	0.28	0.73	0.72	0.00	

TF	Source	Err	Sens	Spec	p-val	Logo
EP300	Cons	0.26	0.84	0.65	0.00	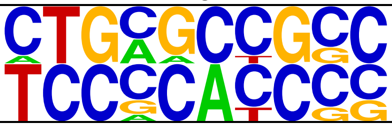
EP300	Cons	0.26	0.80	0.68	0.05	

TF	Source	Err	Sens	Spec	p-val	Logo
MSC	Cons	0.32	0.70	0.67	0.00	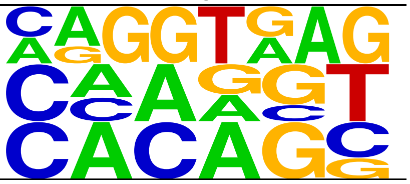
MSC	Cons	0.32	0.67	0.68	0.00	
MSC	Cons	0.33	0.68	0.67	0.00	

TF	Source	Err	Sens	Spec	p-val	Logo
PITX1	Cons	0.25	0.83	0.67	0.00	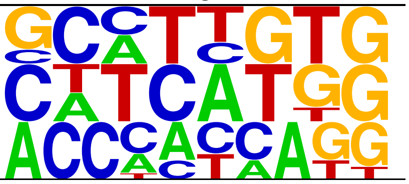
PITX1	Cons	0.25	0.86	0.64	0.00	
PITX1	Cons	0.25	0.86	0.63	0.00	
